# Challenges of benzodiazepine deprescribing in elderly patients attending primary healthcare

**DOI:** 10.3389/fphar.2025.1729081

**Published:** 2026-01-08

**Authors:** Marlon Silva Tinoco, Silvio José Elisei Carvalho Júnior, Aline Istefane de Camargos Ramos, Luciana Soares Rodrigues, Mariana Linhares Pereira, Andre Oliveira Baldoni

**Affiliations:** 1 Universidade Federal de São João del-Rei, São João del Rei, Brazil; 2 Centro Universsitário de Formiga, Formiga - Minas Gerais, Brazil

**Keywords:** ageg, benzodiazepines, deprescribing, primary health care, qualitative research

## Abstract

Benzodiazepine deprescribing in older adults is a complex process influenced by systemic, structural, cultural, and individual factors. This qualitative study examined the challenges faced by primary care professionals during the implementation of a supervised deprescribing protocol in a municipality in Minas Gerais, Brazil. Through thematic content analysis, based on Bardin’s methodology and relevant theoretical frameworks for primary care, interviews with community health workers and nurses were analyzed to identify barriers related to structure, process, and outcomes. The main structural barriers included high physician turnover and lack of trust in healthcare professionals. Process-related problems involved failures in information flow and internal coordination. Challenges related to outcomes and patients included resistance due to prolonged use and fear of withdrawal symptoms, limited understanding of the dose reduction process, missed appointments, and lack of home support. These interconnected factors significantly compromised the effectiveness of the intervention. In summary, this study adds that, within the Brazilian Unified Health System (SUS), stable teams, protected time for medication review, network-based psychiatrists, and home-based logistics are prerequisites for effective deprescribing; without these governance conditions, isolated education and protocols tend to fail. These measures are essential to promote treatment adherence, ensure patient safety, and achieve sustainable results in health services that aim to implement deprescribing.

## 1 Introduction

Population aging is a global phenomenon that has been altering the age structure of societies, with an increase in the proportion of elderly people and a consequent impact on health systems (World Health Organization, 2021). This process is associated with a higher prevalence of chronic diseases, frequent use of multiple medications, and increased polypharmacy—often defined as the simultaneous use of five or more drugs—which increases the risk of drug interactions, adverse events, and reduced therapeutic adherence (Maher et al., 2014; Doherty et al., 2020).

In this context, the practice of deprescribing emerges, understood as the systematic and supervised process of reducing the dose or discontinuing potentially inappropriate medications, based on the continuous reassessment of the risk-benefit ratio for each patient (Bolt et al., 2023; Doherty et al., 2020). Evidence indicates that deprescribing is a safe and beneficial intervention in older adults, capable of reducing adverse events and improving quality of life (McEvoy et al., 2025; Fung et al., 2024; Bloomfield et al., 2020; Zhou et al., 2023; Ibrahim et al., 2021).

Although deprescribing is a clinically safe practice associated with a reduction in adverse events, polypharmacy, and dependence on potentially inappropriate medications, its incorporation into routine primary care still faces multiple challenges. Evidence-based guidelines show that the process is effective and safe when carried out gradually and with adequate clinical follow-up (Pottie et al., 2018; Bloomfield et al., 2020). However, systematic reviews reveal barriers, including limited consultation time, the absence of clear protocols, fragmentation of care, and technical insecurity among professionals, as well as cultural factors such as resistance from patients and families and fear of adverse effects after discontinuation (Okeowo et al., 2023; Doherty et al., 2020).

Recent literature shows that, even in high-income countries, deprescribing medication in elderly patients with polypharmacy faces multiple and persistent obstacles. According to Robinson et al. (2024), in a review encompassing 31 studies, professionals report discomfort in routinely engaging in the deprescribing process, despite recognizing its relevance, due to a lack of specific knowledge, reduced consultation time, fragmented communication, and fear of adverse consequences—including feelings of “abandonment” of clinical care and resistance from patients or caregivers. This evidence reinforces that deprescribing is not just a clinical decision, but depends on organizational, cultural, and systemic factors that shape its feasibility.

A Delphi panel with Brazilian geriatricians identified the main obstacles to deprescribing as insufficient consultation time, communication difficulties between professionals/levels of care, training gaps, as well as fragmentation of care and the need for an institutional culture favorable to continuous therapeutic review (Teixeira et al., 2022). The Brazilian study by Rodrigues et al. (2021), which developed and validated an instrument to assess facilitators and obstacles to benzodiazepine deprescribing in the elderly, highlighted barriers related to resistance from patients and family members, insecurity regarding medication withdrawal, and lack of systematic follow-up by healthcare professionals.

Understanding these barriers requires considering the organizational structure of the Unified Health System (SUS), established by the 1988 Constitution and based on the principles of universality, comprehensiveness, and social participation. The SUS constitutes a broad, decentralized network of public services organized by the federal, state, and municipal levels, with Primary Care—within the scope of Primary Healthcare (PHC)—as the structuring axis for continuous care, prevention of health problems, and coordination of care networks. The Family Health Strategy (ESF), originating from the expansion of the Community Health Agents Program (PACS), has consolidated itself as the main organizational model of PHC by territorializing health actions and promoting longitudinal monitoring of families and communities (Barcellos Ramos, 2024; Paim et al., 2011).

In this arrangement, the nurse plays an essential role in the operationalization of the Family Health Strategy (ESF), acting both in direct care and in managerial, educational, and health surveillance functions. They conduct nursing consultations, comprehensive assessments of health needs, plan team actions, and coordinate care processes, contributing to the unit’s problem-solving capacity and the effective implementation of the principles of comprehensiveness and continuity of care. Their permanent presence in the unit and their proximity to the users’ reality make them a key professional for identifying clinical vulnerabilities, medication-related problems, and needs for therapeutic intervention (Pires et al., 2022).

The community health agent (CHA), in turn, constitutes the fundamental link between health services and the population. Their responsibilities include registering families, regular home visits, actively seeking out at-risk users, developing educational actions, and monitoring priority groups, directly contributing to the reach and effectiveness of primary healthcare. Because they reside or live daily in the assigned area, the CHA identifies situations of vulnerability, hidden needs, access barriers, and care-related problems early on, favoring the building of bonds and the comprehensiveness of health actions (Oliveira et al., 2022).

Given this scenario, understanding the barriers experienced by nurses and community health agents (CHAs) — central actors in primary healthcare (PHC) with complementary roles in the care of older adults—is essential to support strategies for implementing deprescribing protocols that consider the sociocultural, organizational, and structural reality of the Brazilian Unified Health System (SUS). This qualitative study analyzes the perceptions of these professionals during an attempt to implement a benzodiazepine deprescribing protocol for older adults in a small Brazilian municipality, contributing to a contextualized understanding of the factors that hinder or prevent this practice in the daily routine of Brazilian PHC.

### 1.1 Objective

How do nurses and community health workers describe the barriers that hinder the implementation of a benzodiazepine deprescribing protocol for older adults in Primary Healthcare settings in Brazil?

## 2 Methods

This qualitative, exploratory study, part of the project “Effect and feasibility of implementing a benzodiazepine deprescribing protocol,” was developed in Primary Healthcare (PHC) in a municipality in the interior of Minas Gerais, after unsuccessful attempts to implement a Canadian protocol in the real world.

It is important to emphasize that Primary Healthcare (PHC) is the gateway to the Unified Health System (SUS), the Brazilian public health system, and its objective is to resolve most of the population’s health needs through integrated actions in defined territories, through actions of promotion, prevention, diagnosis, treatment and rehabilitation of individual, family and collective health problems (Barcellos Ramos, 2024; Paim et al., 2011).

The Canadian protocol calls for the gradual withdrawal of benzodiazepines in elderly patients using them for isolated insomnia, with a 25% dose reduction every 14 days, and with 12.5% dose reductions in the final reductions to avoid signs and symptoms of benzodiazepine withdrawal (Pottie et al., 2018). The research group then conducted on-site training with primary healthcare teams throughout the municipality. These teams are composed of a family physician, a nurse, and community health workers (who are mid-level workers who reside near health units and are trained to be a link between patients and health professionals; their main functions include conducting home visits, providing guidance and monitoring the population on health and disease prevention, and identifying and mapping health problems in the territory). During the protocol’s implementation, patients were identified by doctors, nurses, and community health agents (CHAs). They were invited to participate in the deprescribing process during initial assessment with a nurse, during a medical consultation, or at home by a CHA. If they agreed to participate, they underwent an assessment with a nurse to explain the importance of the project and learn sleep hygiene measures, followed by a medical consultation to reduce their weekly dose of benzodiazepine. After initial training sessions covering the rational use of benzodiazepines and the entire deprescribing protocol (reducing doses every 14 days, identifying and managing withdrawal signs and symptoms), the research team followed up with weekly visits to the units and through telephone contact and instant messaging applications. However, the teams were unsuccessful in recruiting and attending to elderly patients using benzodiazepines who were served by the municipality’s primary healthcare services.

For this study, four nurses working in primary healthcare units and nine community health workers working in these units were selected and invited to participate. All professionals have direct contact with patients using benzodiazepines and participated in training sessions conducted by the research group regarding the Canadian benzodiazepine deprescribing protocol proposed by Pottie et al. (2018).

The interviews were conducted between October and December 2024, in a reserved space within the health units, ensuring privacy, confidentiality, and comfort for the participants. Each session lasted an average of 25 min and followed a semi-structured script developed from a prior review of the literature and evidence from the implementation trial. The script was organized into thematic blocks that addressed: (i) barriers related to patients; (ii) barriers related to work routine; (iii) barriers related to the professional (Annex I).

The interviews were conducted by a single trained researcher to ensure standardization in application and neutrality in conduct. All material was audio-recorded using a smartphone, with the prior consent of the participants, and subsequently transcribed in full, preserving pauses, emphases, and colloquial expressions relevant to the meaning of the speech. The transcriptions were double-reviewed and coded using analytical memos to avoid loss of semantic and contextual nuances.

The analytical process followed Bardin’s (2016) Content Analysis method, based on three interdependent phases:

1. Preliminary analysis: this consisted of organizing the corpus, a cursory reading of all interviews and field notes, identification of context and recording units, and formulation of guiding analytical questions, such as: “What structural and organizational factors hindered the application of the deprescribing protocol?” and “How do professionals perceive the role of multidisciplinary teams in this process?”.
2. Exploration of the material: the transcripts were subjected to open coding, in which two researchers coded the recording units (complete ideas). Following this, a consensus review was conducted, and the data were grouped into thematic categories and subcategories, guided by reflections on the care of users of primary healthcare units in Brazil, using Donabedian’s (2003) framework, in order to structure the evidence along the axes of “structure,” “process,” and “outcomes.”
3. Treatment, inference, and interpretation: In this stage, the data were triangulated with field notes and observational records collected during the deprescribing trial, allowing the identification of convergences and divergences between professionals’ perceptions and observed situations. The final categories were interpreted in light of Donabedian’s theoretical model, highlighting the relationships between structural conditions (instability of bonds, precarious logistics), work processes (interprofessional communication, adherence to the protocol), and expected results (feasibility of deprescribing in the real world).

This approach ensured methodological rigor and internal validity, as advocated by Bardin. The triangulation of sources—interviews, field notes, and records of the implementation process—allowed for increased reliability of inferences and a deeper understanding of the phenomena studied, as recommended by Santos et al. (2020).

The field diary was initiated during the team training and concluded with the qualitative interviews conducted with the professionals after the process. The diary helped to identify and define the nurses and community health agents as interview participants.

Data collection and analysis were performed simultaneously, which ensured a more consistent interpretation of the results and allowed for the appropriate restructuring of data collection according to each assessment, thus favoring the validation of the research.

## 3 Results

Of the 4 nurses invited, 3 accepted and participated, and 1 professional did not participate because they were on medical leave. All 9 community health agents invited who were in the units on the dates of the interviews were invited and participated. The interviews were categorized according to the proximity and similarity of the themes.

The categories and core meanings emerged from the interviews and were separated by professional category. In accordance with the analytical procedures, the results were organized according to Donabedian’s tripartite model—structure, process, and outcomes—in order to ensure theoretical coherence and reflect how the coded data converged on distinct thematic domains. During the categorization stage, each code and subcode generated by Bardin’s content analysis was examined for its semantic approximation to the components of Donabedian’s model. The structural categories included contextual and organizational conditions that shaped the feasibility of deprescribing (e.g., workforce instability, shortage of support staff). The process-related categories encompassed barriers inherent to workflow, communication, and team coordination practices (such as workload distribution, limitations on home visits, and failures in internal information flow). The outcome categories referred to the consequences perceived by professionals, especially those related to patient engagement, adherence, and behavioral responses during the deprescribing attempt. This organization into structure-process-outcome allowed for a more explicit demonstration of how systemic and operational conditions interacted to shape the overall viability of the intervention. Regarding the interviews with the Community Health Agents (CHAs), although they were within the context of the Donabedian triad, categories emerged not only related to the health service, but also to the patient and social capital. All categories, as well as their core meanings, are presented in Table 1.

**TABLE 1:** Categories and Core Meanings emerging from interviews conducted with nurses and community health agents (CHAs) in the context of identifying barriers to the deprescribing process of benzodiazepines in elderly people, Brazil, 2025.

Professional	Category	Core meanings
Nurses	Structural obstacles	High turnover of medical professionals; lack of human resources
	Procedural obstacles	Nurse overload; unavailability for home visits; limitations related to community health workers (CHAs)
	Obstacles to results	Absenteeism; resistance to change; culture of self-medication; professional motivation
Community health workers	Challenges related to the health service	High turnover of medical professionals; lack of connection with doctors; failures in information flow and internal coordination
	Patient-related challenges	Absenteeism; perception of absence of risks; dependence
	Challenges related to social capital	Insufficient home support for the elderly

### 3.1 Content analysis of interviews with nurses

#### 3.1.1 Structural obstacles

##### 3.1.1.1 High turnover of medical professionals

Nurses reported that the constant turnover of doctors compromises continuity of care, leads to loss of rapport, and weakens the implementation of the protocol.

E2: “Doctor change. John 1 left, John 2 came. John 2 left, Mary 1 came. Mary 1 left, Mary 2 came. Mary 2 left, Mary 3 came.”

E1: “A different doctor arrives every hour, some did not even know about the project (deprescribing protocol).”

E3: “But then, as the weeks and months went by, this constant switching happened. There was 1 year when I had five different doctors in 1 year. Then the next doctor would come in and say, ‘Oh no, keep taking the medication.”

Throughout the training period, constant changes in prescribers were reported, and training was provided for each new doctor who joined the teams.

E2: “Without a competitive selection process, professionals change every month, preventing the development of a bond with the patient.”

E2: “So here we only have two permanent doctors, Maria 4 and João 3. But the rest are all on temporary contracts. And with these temporary contracts, they stay until they find a better offer. So, it's a major obstacle.”

##### 3.1.1.2 Lack of human resources

The shortage of human resources for administrative tasks overburdens and compromises the clinical and care management activities of nurses, hindering the organization of educational actions and monitoring of benzodiazepine use.

E1: “I was left without a receptionist, I already lose one agent (community health agents (ACS)) for the day.”

E1: “Maternity leave, vacation, sick leave. it’s just us left to handle it all.”

E2: “The scale never closes, and that makes it impossible to work on a new project.”

E3: “Today, for example, there is not one, there’s only one agent here.”

#### 3.1.2 Procedural obstacles

##### 3.1.2.1 Nurse overload

The nurses reported an excess of care and management responsibilities, hindering their ability to dedicate time to deprescribing. During the implementation of the protocol, upon arriving at the units, the nurses encountered queues for preventive exams, vaccinations, COVID-19 tests, numerous dengue cases requiring triage, among other demands.

E2: “… if we had a lower demand from patients, not only in the deprescribing of benzodiazepines, but also in other lines of care, such as hypertension, diabetes, and cervical and breast cancer, we would be able to provide better assistance.”

E1: “Let’s focus on prevention. So, in the first week, focus a lot on prevention, and in the next week, give them another task. So, sometimes we end up accumulating a lot of things for them (the Community Health Agents), right? Things they do not talk about, you know? I think we need more. the nurse is present, diligently there, motivating, checking in every day.”

E2: “We have several government programs that we have to manage, right? Prenatal care, preventative care, vaccinations, right? And in vaccination campaigns, we have to supervise the health agents, handle the caregiving, the management of the unit, the epidemiological investigation … it's an accumulation of functions for the nurse. And with this accumulation of functions, we ‘re almost like we’re putting out fires … ”

E3: “Because our service does not stop, it demands time.”

E3: “There’s the constant demand at the gas station, Monday through Friday, you know? So dedicating more time to that becomes complicated. What became complicated was intertwining all these things.”

##### 3.1.2.2 Unavailability for home visits

Because this process involves the care of elderly individuals, some of whom are confined to their homes and have difficulty accessing healthcare facilities, it would be beneficial to conduct periodic home visits (every 15 days) to discontinue prescriptions. However, difficulties in obtaining transportation restrict home visits for monitoring these patients.

E2: “We have difficulties because we rely on the city’s transportation to make visits. We have a car, one afternoon a week for me and the doctor to share. (…) So, it's from 1:00 to 4:00 in the afternoon for me and the doctor to share (…) so, it's complicated (…)”

##### 3.1.2.3 Limitations related to community health workers (CHWs)

There was a complaint during the implementation of the deprescribing protocol that community health workers (CHWs) were unable to recruit or had low engagement when the nurse approached them to inquire about patients eligible for deprescribing.

E1: “The agents thought it would not work, they could not explain it to the patients.”

E2: “They were afraid to interfere, afraid of being held responsible.”

The research team, while implementing the deprescribing protocol, repeatedly inquired about newly identified patients, but almost all responses were negative (they did not know about the patients, they could not find them in a single attempt to visit their homes).

E1: “They themselves did not really believe it, so the patient did not believe it either.”

E3: “They (community health workers) also use [benzodiazepines]…and can’t stop.”

## 4 Obstacles to results

### 4.1 Absenteeism

Nurses reported that many patients do not attend appointments or abandon follow-up care. This absenteeism seems to reflect the overall context of structural and process-related difficulties within healthcare units, resulting in a lack of trust and connection, and discouraging patients from attending the unit.

E3: “And many patients, we would sometimes schedule appointments, the patient wanted to see them, but they would not show up, they would not come to the clinic.”

E1: “Yes. Sometimes they make plans and then do not show up.”

E2: “But then the patient would start missing appointments, disappear, never show up again…”

E1: “You (patient) go to the unit where the doctor needs to reduce your medication, but you do not come. Because you did not go (patient’s name) on the day you were supposed to, the doctor was going to adjust your medication. Then I’ll get the prescription for myself, ask her to reduce it there.”

### 4.2 Resistance to change

Resistance to discontinuing benzodiazepines emerged as one of the main obstacles. Professionals frequently complained about patients’ initial resistance to discontinuing benzodiazepines, with some even refusing to listen to more detailed explanations about the discontinuation protocol. This resistance may be linked to fear of insomnia upon stopping the medication, lack of trust in the support team during the process, among other factors.

E3: “Yes, I think the person shows resistance. An example I have at my work here (…) Our patients, who are lovely. They can’t do it.”

E1: “You understand? She‘s so used to it, so dependent on the medication, just talking about taking it away is shocking.”

E3: “…one is the resistance to stopping the medication, and another is that sometimes they do not see much benefit.”

E3: “Because of the addiction, they do not, they do not, they do not quit; this addiction is harmful.”

### 4.3 Culture of self-medication

Nurses reported that sharing medication within the family perpetuates misuse.

E1: “One of the family members takes it, then one sleepless night when the son had problems at work, argued with his girlfriend, the mother is already giving it to him to take. The next day he’s already coming here asking for a prescription. (*…*) the mother comes to get the prescription because her medicine did not last 30 days and she‘s sharing it with the rest of the family.”

### 4.4 Professional motivation

Failures in proactive outreach and team demotivation in following the deprescribing protocol were identified. Nurses reported during the process that they had difficulty maintaining motivation in the face of challenges, not only in executing the deprescribing protocol but also in dealing with the demands that overwhelmed them.

E1: “There was a lack of active searching for this patient at home. So, I think the execution was a little flawed in that respect.”

E3: “It's a bit of complacency. Not wanting to change the routine, the way things are, it's fine. You understand?”

E1: “Maybe it's that lack of motivation, of thinking it's important, that it will yield results, you know?”

E1: “The management aspect discouraged me because I realized I was not going to be able to handle it. You understand? And I saw that she (the doctor) was all about prescriptions, that part of her project was all about prescriptions.”

### 4.5 Content analysis of interviews with community health workers (CHWs)

#### 4.5.1 Challenges related to the health service

##### 4.5.1.1 High turnover of medical professionals

The community health agents (CHAs) reported that the frequent change of doctors disorients patients and disrupts care strategies. This point was also central to the perspective of the nursing professionals, and the reason for the need for new training by the research team with each change of prescriber.

ACS2: “The turnover of doctors here is high, is not it? … we ended up training several doctors, and then new ones came in.”

ACS7: “Because one person starts something and when the other (doctor) arrives, it's already different.”

ACS3: “There have been several doctors here, there’s a fairly high turnover, one starts something and when another arrives it's already different.”

##### 4.5.1.2 Lack of connection with doctors

Reports have shown that patients have more confidence in specialist physicians working in supplementary roles within the private healthcare network, especially psychiatrists, than in primary care professionals. It is important to emphasize that in the Brazilian healthcare system, specialists are accessed only after referral from the primary care team, which is the entry point to the system. These professionals work in medical specialty centers and/or private practices.

ACS6: “…because I’ve heard this before: ‘the doctor wants to take away my medication that my psychiatrist prescribed, I’ve been taking it for so many years, she’s going to mess with my medication’.”

ACS6: “My psychiatrist prescribed it for me, and the doctor wants to take it away… there’s a lot of that.”

##### 4.5.1.3 Failures in information flow and internal coordination

The community health agents (CHAs) identified communication failures between teams and loss of information during the process. This is strange, since all teams had undergone at least two training sessions on the deprescribing protocol and received monthly visits from the research group to support the process.

ACS8: “I had not received a list of where we had gone… so I think it got lost along the way, nobody, I think, stopped, because they were not called.”

ACS9: “To tell you the truth, nobody knows, it got lost, they did not pass it on to us, you know?”

ACS2: “Some community health agents did not even know, they did not get involved.”

## 5 Patient-related challenges

### 5.1 Absenteeism

Community health workers (CHWs) reported that many patients do not attend visits or give up on the process. Even patients who signed the consent form to participate in the project did not show up at the unit to begin the gradual withdrawal of benzodiazepines.

ACS6: “The person said they were going to come, but then they did not want to get involved, they think they will not be able to handle it… they were not interested.”

ACS1: “Some patients we scheduled and they did not show up… They gave up, right? They gave up.”

ACS6: “In my case, they said they were coming here. No… they did not show up, they did not even come here to find out… I called again, they did not want to come.”

ACS5: “I even scheduled an appointment, but they did not show up … Then they told me: ‘I’m not going to try right now because I’m going through a difficult time. We’ll schedule it later,’ but that ‘later’ never comes.”

### 5.2 Perception of absence of risks

The reports revealed a fear of change, familiarity with the medication, and a perception of the absence of adverse effects.

ACS5: “I’m not feeling any reaction. I’ve already tried other medications, they did not work.”

ACS6: said, “ I do not feel anything with him, like, he’s not hurting me because I sleep well with him, he’s not bothering me at all.”

ACS6: “They were saying, ‘I do not feel anything with him,’ they were not understanding the risks of memory loss and falls that we explained.”

### 5.3 Dependence

Community health workers (CHWs) reported that patients believe they depend on benzodiazepines to sleep.

ACS1: “It’s insomnia, really. Yes, insomnia, plain and simple.”

ACS6: “They would say things like, ‘I can’t quit,’ some tried for a few days and came back… They’ve even said directly: ‘I can’t do it.”

ACS3: “I can’t sleep, I can’t go without it, it causes anxiety, it causes crises and everything else.”

ACS6: “She came here, talked to the nurse, to the doctor, scheduled an appointment and everything… Then she said she was n’t able to, and asked to come back.”

ACS4: “They thought they would not be able to quit, that they depended on the medication to sleep.”

ACS6: “Some even tried, they came here, talked to the nurse and the doctor, made an appointment and everything, but… they tried for a few days and came back.”

### 5.4 Challenges related to social capital

#### 5.4.1 Insufficient home support for the elderly

Agents reported that the lack of home support makes it difficult to adhere to the deprescribing process.

ACS9: “Very elderly people… have difficulty even sleeping, not just taking their medication on their own, you know? … The person is alone, without someone there to guide them at home.”

ACS8: “She did not understand… the amount to reduce, how she was going to take it…”

ACS7: “For a person alone, without someone to support them… it’s not so easy to make that change.”

ACS2: “The family likes the person to take it because it makes them calmer.”

ACS7: “The family members said: if you take away the medication, she will not sleep, it will cause problems.”

#### 5.4.2 Comparison between nurses and community health workers (CHWs)

**FIGURE 1:**
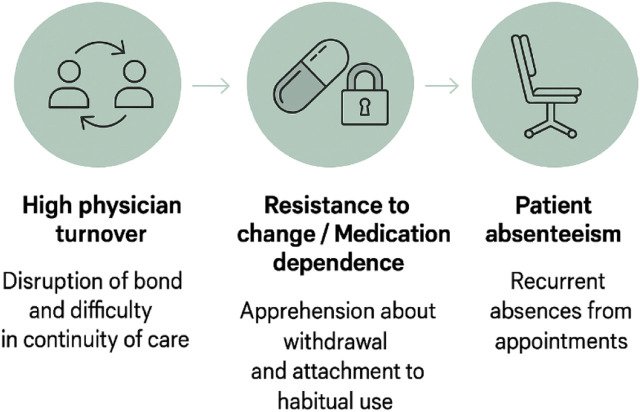
Common barriers to the deprescribing process in older adults, as perceived by nurses and community health workers (CHWs). Source: Author’s own elaboration.

Both nurses and community health workers (CHWs) identified the high turnover of medical professionals as one of the main obstacles, in addition to pointing to patient absenteeism and resistance, perception of absence of risks and dependence on medication as central barriers to the deprescribing process (Figure 1).

Among nurses, aspects related to the lack of human resources and the nurse’s work process also stood out—including the unavailability for home care and limitations related to health agents—as well as outcome factors, such as a culture of self-medication and professional motivation.

Among the community health agents, issues related to the lack of connection with doctors, failures in the flow of information and internal coordination, the perception of a lack of risk on the part of patients, and the inadequacy of home support for the elderly emerged more strongly.

These findings demonstrate that, although each professional category experiences specific obstacles in their daily work, there is a common core of structural and behavioral barriers that directly impacts the success of deprescribing.

## 6 Discussion

Content analysis revealed a comprehensive set of barriers to benzodiazepine deprescribing in older adults in primary healthcare, organized into categories that express distinct perceptions among nurses and community health workers (CHWs), but which share common core elements, mainly related to structural and behavioral factors.

### 6.1 Structural obstacles (nurses)

The high turnover of medical professionals was unanimously highlighted by nurses as one of the main obstacles to the implementation of the protocol, as it compromises the continuity of care, weakens the therapeutic bond, and disrupts previously established strategies. These effects have already been described in national studies, which associate team instability with loss of longitudinality and weakening of trust relationships between professionals and users in Primary Care (Oliveira et al., 2024; Campos and Malik, 2008). In the context of deprescribing, the influence of the attending physician and the need for discussion and follow-up are determining factors in patients’ willingness to discontinue medication (Reeve et al., 2016).

The scarcity of human resources—especially administrative support and community health agents (CHAs) — has led to an overload of nurses, reducing the time available for actions necessary for the development of the deprescribing process. This scarcity has generated overload among nurses, who accumulate care and managerial activities. A qualitative study with primary healthcare nurses showed that the lack of support staff and the management deficit in the units result in an accumulation of tasks and physical and emotional exhaustion, affecting the continuity of actions and job satisfaction (Saraiva Felix et al., 2023).

### 6.2 Procedural obstacles (nurses)

Among the procedural obstacles, the accumulation of duties by nurses stood out, as they simultaneously perform care, management, surveillance, and supervision of community health agents (CHAs), hindering continuous involvement with deprescribing protocols. Peat et al. (2022) identified that time constraints during consultations and workload pressures are barriers frequently reported by primary care professionals, who often prioritize other clinical demands over medication review.

The unavailability of transportation for home visits restricted the reach of the intervention, especially for elderly people with mobility limitations. This fact hinders these elderly people’s access to services, since home visits promote humanized care, create bonds between professionals and the elderly, improve the quality of life of the population, and implement health actions focused on the care of the assisted population (Quirino et al., 2020; Silva et al., 2016).

Technical limitations and insecurity among some community health workers (CHWs) in supporting the deprescribing process have also been reported, either due to lack of training or personal use of benzodiazepines. Well-trained community health workers play a strategic role in mediating between the team and the community, and their technical confidence directly impacts patient adherence (World Health Organization, 2020).

### 6.3 Obstacles to outcome (nurses)

Among the challenges related to outcomes, nurses highlighted absenteeism, resistance to change, a culture of self-medication, and professional demotivation. Absenteeism impairs continuity of care, the effectiveness of services, and the planning and organization of health services (Silva et al., 2021). The occurrence of absenteeism possibly reflects inequalities in access to and equity of services, as well as the fragmentation of care in the public system, which, although prioritizing areas such as pharmaceutical assistance and maternal and child health, still presents gaps in the integration of care (Machado, 2018).

Patients’ resistance to discontinuing benzodiazepine use is associated with prolonged use, the perception of subjective benefits (such as improved sleep and anxiety relief), and fear of relapse or worsening of symptoms. Users describe these medications as indispensable for wellbeing and express insecurity regarding withdrawal, especially due to fear of withdrawal and distrust in therapeutic alternatives (Pétein et al., 2024; Burmester et al., 2024; Reid Finlayson et al., 2022). Furthermore, recent studies indicate that beliefs about the continued need for medication and fear of symptom recurrence after discontinuation constitute persistent barriers to deprescribing (Robinson et al., 2024).

The culture of self-medication is a phenomenon described in Brazilian reality and is characterized by a behavior that affects different economic and social classes. This practice leads to a large network of misinformation regarding the rational use of medications and the adverse reactions caused by them (Borges et al., 2023; Fernandes and Cembranelli, 2015).

The demotivation of nurses in Primary Healthcare stems from multiple structural and organizational factors. Work overload, precarious employment, accumulation of functions, and lack of institutional recognition reduce the autonomy and engagement of these professionals, favoring burnout and compromising the quality of care (Alvarenga and Sousa, 2022; Pereira et al., 2023).

### 6.4 Obstacles related to the health service (ACS)

Community health workers (CHAs) highlighted institutional and organizational difficulties that directly impacted the implementation of the intervention.

High physician turnover, from the perspective of community health agents (CHAs), was perceived by patients as a discontinuity of care, generating distrust in new approaches. The lack of a bond between patients and primary care physicians was a significant barrier, especially in cases of patients who had been followed by psychiatrists for years. Patients tended to resist the proposal of deprescribing medication by professionals with whom they did not have a history of care. The continuity of the doctor-patient relationship is a determining factor for the rational use of medications and the success of deprescribing. Lasting bonds strengthen trust, promote dialogue, and allow for safer therapeutic decisions, resulting in fewer inappropriate prescriptions and greater adherence to medication withdrawal strategies (Te Winkel et al., 2023; Ailabouni et al., 2016).

Ineffective communication between professionals and a lack of coordination between levels of care are significant barriers to deprescribing, leading to loss of information and disarticulation of actions. The fragmentation of information and the absence of continuity of care compromise therapeutic review and hinder shared decisions about medication use (Peat et al., 2022; Robinson et al., 2024).

### 6.5 Patient-related challenges (PACs)

Community health workers (CHWs) provided complementary insights to those of the nurses by highlighting barriers centered on patients’ behavior and beliefs:

Absenteeism was also strongly reported, with patients dropping out of the process or failing to attend scheduled appointments, which was a major obstacle to the execution and organization of the service (Silva et al., 2021).

The perception of a lack of risk was recurrent: many elderly people stated that they “did not feel anything wrong” with the prolonged use of benzodiazepines, failing to recognize the risks present in lists of potentially inappropriate medications for the elderly, such as cognitive deficits and falls associated with chronic use (American Geriatrics Society Beers Criteria® Update Expert Panel, 2023; O'Mahony et al., 2023).

Dependence was frequently mentioned, with patients reporting being unable to sleep or perform their activities without the medication—a phenomenon widely described in the literature (American Geriatrics Society Beers Criteria® Update Expert Panel, 2023; O'Mahony et al., 2023; Pottie et al., 2018).

### 6.6 Obstacles related to social capital (SCC)

Finally, community health agents (CHAs) highlighted the lack of home support as a central barrier, especially among older adults who live alone or have fragile support networks. The presence of engaged family members or caregivers promotes adherence to the therapeutic plan, allows for the monitoring of possible adverse effects, and expands dialogue with the health team. Conversely, the absence of home support or fragile support networks represents a significant barrier, particularly among older adults who live alone or have functional limitations. Studies indicate that family involvement and health education are decisive factors in strengthening confidence in the deprescribing process and promoting a safer and more collaborative approach (Teixeira et al., 2022; Krüger et al., 2025).

### 6.7 Synthesis

A comparison between the emerging categories of nurses and community health workers (CHWs) shows complementarity: while the former emphasize structural and organizational aspects of the work process, the latter highlight relational, communicational, and socio-behavioral barriers manifested in daily interaction with patients and families. Both perspectives converge on the need for multilevel approaches that combine structural interventions (team stability, clear protocols), procedural interventions (improvement of communication flows), clinical interventions (professional training), and social interventions (health education and family involvement).

Analysis of the findings reveals that barriers to deprescribing emerge interdependently across the three dimensions proposed by Donabedian (2003) — structure, process, and outcome. Structurally, insufficient human resources, high physician turnover, and organizational weaknesses compromise continuity of care and coordination among professionals. In the process, ineffective communication among team members, fragmented information flow, and the absence of consolidated clinical protocols stand out, hindering shared decision-making and therapeutic monitoring. Finally, in the outcome, patients’ limited perception of the risks of prolonged benzodiazepine use and team demotivation negatively impact adherence to deprescribing strategies and the safety of care. Thus, the integration of adequate structure, effective communication processes, and outcomes focused on patient education and engagement is fundamental to strengthening the practice of deprescribing in Primary Healthcare.

### 6.8 Limitations and strengths of the study

This study has some limitations. Because it is a qualitative investigation conducted in a single municipality, the transferability of the findings to other contexts may be limited. The analysis focused on the perceptions of nurses and community health agents, without including physicians, pharmacists, managers, or patients, thus restricting the scope of perspectives on the deprescribing process.

On the other hand, the study has important strengths. It offers an in-depth analysis of the perceptions of key professionals, often neglected in studies on deprescribing, applying a solid theoretical framework. The findings show internal coherence and convergence between different professional categories, reinforcing their validity. Furthermore, they provide practical and contextual support for public policies and local interventions aimed at implementing deprescribing practices in primary healthcare.

## 7 Conclusion

This study deepens the understanding of barriers to benzodiazepine deprescribing in the elderly by highlighting, in a contextualized manner, how structural, organizational, and sociocultural characteristics specific to Brazilian Primary Care (especially in small municipalities) directly interfere with the viability of clinical protocols developed in other health systems. In contrast to part of the international literature, which describes obstacles predominantly related to patients’ perceptions and professionals’ time constraints, our findings show that, in the Brazilian Unified Health System (SUS), factors such as high physician turnover, weak care relationships, insufficient human resources in the minimum team, failures in information flow, nurse overload, and logistical limitations for home care constitute structural barriers that can prevent even the beginning of the deprescribing process.

Furthermore, the study demonstrates that community health agents (CHAs) and nurses experience distinct, yet complementary, barriers: while nurses describe organizational obstacles that are decisive for the execution of the protocol, CHAs highlight socio-behavioral obstacles related to users, such as low risk perception, medication dependence, and the fragility of home support—elements that are rarely explored in such detail in national studies. By integrating these two professional perspectives, the study contributes to broadening the understanding of the conditions necessary for the implementation of deprescribing in Brazilian Primary Healthcare, emphasizing that evidence-based interventions tend to fail when there is no team stability, continuity of care, and effective internal coordination. Thus, the findings reinforce the importance of strategies that combine organizational changes, strengthening of multidisciplinary work, and educational actions aimed at users, in order to make deprescribing feasible in the daily routine of Primary Healthcare.

## Data Availability

The original contributions presented in the study are included in the article/Supplementary Material, further inquiries can be directed to the corresponding authors.
