# Supplementary material for: Challenges of benzodiazepine deprescribing in elderly patients attending primary healthcare
Source: Front Pharmacol. 2026 Jan 8;16:1729081. doi: 10.3389/fphar.2025.1729081 (PMC12823946; doi:10.3389/fphar.2025.1729081)
Supplement: Supplementary file 1 [file Supplementaryfile1.docx]

**Annex I - Semi-structured questionnaire for qualitative investigation of the obstacles to the deprescribing process from the perspective of primary health care professionals.**

The project didn't go as planned, I wanted to hear a little bit about it from you.

Why do you think so few patients were treated in this project here at the unit?

Patient-related issues

1. How motivated were the patients when invited to come to the unit to discontinue their prescriptions?
2. Regarding patient behavior, how do you view it?

For this, we suggest home visits: what do you think of this option?

1. What happened when patients with scheduled appointments didn't show up?
2. After several attempts, the research team recruited patients who agreed to participate and scheduled appointments with the community health agents. What was the patients' reaction after this recruitment?

Work routine

1. Regarding the routines of the other professionals, how do you think they acted in this process?
2. We were told there was a high turnover of doctors in the department, what can you tell me about that?
3. We started the project at the end of the Covid-19 pandemic. How did this influence the unit's routine? And how did dengue fever affect the professionals' routine?

And to the professional

1. Regarding yourself, did you feel motivated?
2. Regarding your work routine, what can you tell me?

But considering that it didn't work out, can you identify any factor that might have hindered your performance?
